# Proteins of the Ciliated Protozoan Parasite *Ichthyophthirius multifiliis* Identified in Common Carp Skin Mucus

**DOI:** 10.3390/pathogens10070790

**Published:** 2021-06-22

**Authors:** Mona Saleh, Abdel-Azeem S. Abdel-Baki, Mohamed A. Dkhil, Mansour El-Matbouli, Saleh Al-Quraishy

**Affiliations:** 1 Clinical Division of Fish Medicine, University of Veterinary Medicine, 1210 Vienna, Austria; Mansour.el-matbouli@vetmeduni.ac.at; 2 Zoology Department, College of Science, King Saud University, Riyadh 11451, Saudi Arabia; aabdelbaki@science.bsu.edu.eg (A.-A.S.A.-B.); mdkhil@ksu.edu.sa (M.A.D.); squraishy@ksu.edu.sa (S.A.-Q.); 3 Zoology Department, Faculty of Science, Beni-Suef University, Beni-Suef 62521, Egypt; 4 Department of Zoology and Entomology, Faculty of Science, Helwan University, Cairo 11795, Egypt

**Keywords:** aquaculture, ciliate, common carp, ichthyophthiriosis, infection, proteomics

## Abstract

The skin mucus is the fish primary defense barrier protecting from infections via the skin epidermis. In a previous study, we have investigated the proteome of common carp (*Cyprinus carpio*) skin mucus at two different time points (1 and 9 days) post-exposure to *Ichthyophthirius multifiliis*. Applying a nano-LC ESI MS/MS technique, we have earlier revealed that the abundance of 44 skin mucus proteins has been differentially regulated including proteins associated with host immune responses and wound healing. Herein, in skin mucus samples, we identified six proteins of *I. multifiliis* associated with the skin mucus in common carp. Alpha and beta tubulins were detected in addition to the elongation factor alpha, 26S proteasome regulatory subunit, 26S protease regulatory subunit 6B, and heat shock protein 90. The identified proteins are likely involved in motility, virulence, and general stress during parasite growth and development after parasite attachment and invasion. Two KEGG pathways, phagosome and proteasome, were identified among these parasite proteins, mirroring the proteolytic and phagocytic activities of this parasite during host invasion, growth, and development, which represent a plausible host invasion strategy of this parasite. The results obtained from this study can support revealing molecular aspects of the interplay between carp and *I. multifiliis* and may help us understand the *I. multifiliis* invasion strategy at the skin mucus barrier. The data may advance the development of novel drugs, vaccines, and diagnostics suitable for the management and prevention of ichthyophthiriosis in fish.

## 1. Introduction

The ciliated protozoan parasite *Ichthyophthirius multifiliis* is one of the highly virulent pathogens in freshwater aquaculture and ornamental fish industries. It infects the skin and gill epithelia of virtually all species of freshwater fish. Among the main disease characteristics of fish infected with ciliates are black coloration; increased production of mucus; damage of scales; hemorrhagic, decolorized areas on the skin; and dermal necrotic lesions that ultimately damage tissues leading to high mortalities [1]. It has been proposed that theronts invade fish epithelia via moving between two cells [2,3]. Because exactly at this site the mucus cells are exposed to the fish surface, the invasive stages apparently get access to the epidermis by entering mucous cells and in this way stimulate the mucus secretion [4,5]. This was potentiated by noticing that theronts were attracted and chemotactically reacting to serum constituents in fish mucus [6]. Pathogen invasion can elicit immune reactions in the skin mucosal barrier, and fish that endure a primary infection develop resistance to re-infection [7]. Various approaches including immunoprophylaxis and chemotherapeutics were applied for disease management. Yet, none of those methods turned into a great success, and no vaccine to control the disease triggered by *I. multifiliis* successfully had been reported [7,8,9]. Hence, it is crucial to monitor novel drug and vaccine opportunities for the parasite. A profounder knowledge of host parasite communication at the spot of infection may also help the development of effective management tools against *I. multifiliis* [9].

The carp skin mucus exhibits key functions in stimulating vital immune reactions that are necessary for defending fish tissues against self and parasite proteases. Actually, the protein content of fish skin mucus provides its anti-inflammatory and protective characteristics. Indeed, proteomic analyses reveal proteins related to virulence, which are produced by invaders to conquer the fish host’s immune responses and to assess the composition of the protein of different fish tissues in response to environmental stressors and diseases [10]. For instance, the infection with the ectoparasite sea lice (*Lepeophtheirus salmonis*) results in excessive mucus secretion from fish skin. The alterations in the protein profile of Atlantic salmon skin mucus were assessed using proteomic analysis in response to sea lice. The identified protein component of the skin mucus included intracellular proteins, calmodulin, actin, hemopexin, plasma proteins (apolipoproteins, lectin, plasminogen, and transferrin), and ß-actin, which is the significant component [11].

Recently, the common carp skin mucus proteome profile has been assessed at 1 day and 9 days after infection with *I. multifiliis* [5,12]. A variety of proteins were differentially modulated. Several proteins essential for metabolism were upregulated, whereas multiple downregulated proteins were predominantly structural. Additionally, novel proteins were detected and could act as possible biomarkers for injuries caused during parasitic infection such as olfactomedin 4, lumican, dermatopontin, papilin, and I cytoskeletal 18 [12]. In addition, the abundance of 19 immune-related and signal transduction proteins was altered, comprising the epithelial chloride channel protein, galactose specific lectin nattection, high choriolytic enzyme 1, lysozyme C, granulin, and protein-glutamine gamma-glutamyltransferase 2 [5]. Moreover, several lectins and a varied collection of different serpins with protease inhibitory characteristics were recognized. These apparently activate the lectin pathway and regulate proteolysis, increasing the carp innate immunity and providing defensive features of the skin mucus of fish [5].

Understanding host-pathogen interactions may enable illuminating key regulators and invasion mechanisms of the parasite and fish protective strategies that can pave the way for further studies aimed at developing novel drugs for disease control in aquaculture. In the present study, we identified proteins of *Ichthyophthirius multifiliis* associated with skin mucus in infected common carp.

## 2. Results and Discussion

**Figure 1:**
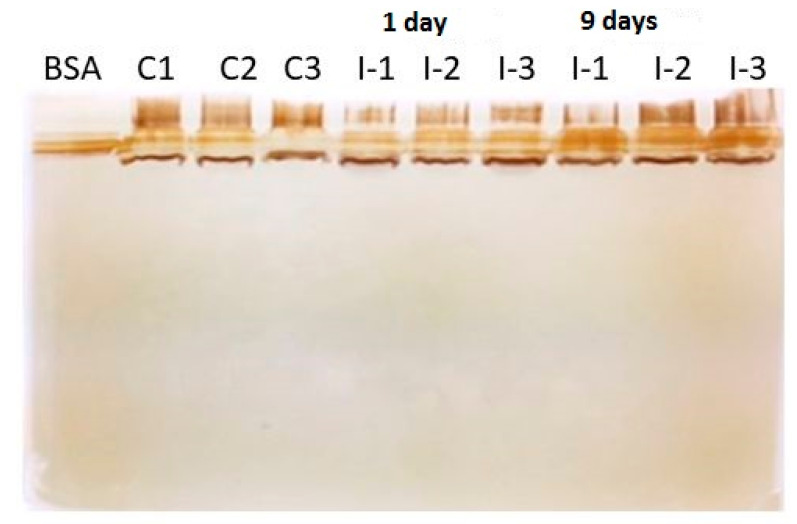
Silver stained 1D-gel of carp mucus protein samples. BSA: bovine serum albumin, C1–C3: control mucus samples, and I-1-3: Ichthyophthirius multifiliis-infected carp mucus samples at 3 and 9 days post exposure.

Following protein separation using SDS-PAGE (Figure 1) and in-gel digestion [13,14], six proteins of *I. multifiliis* were identified in infected carp skin mucus.

**Figure 2:**
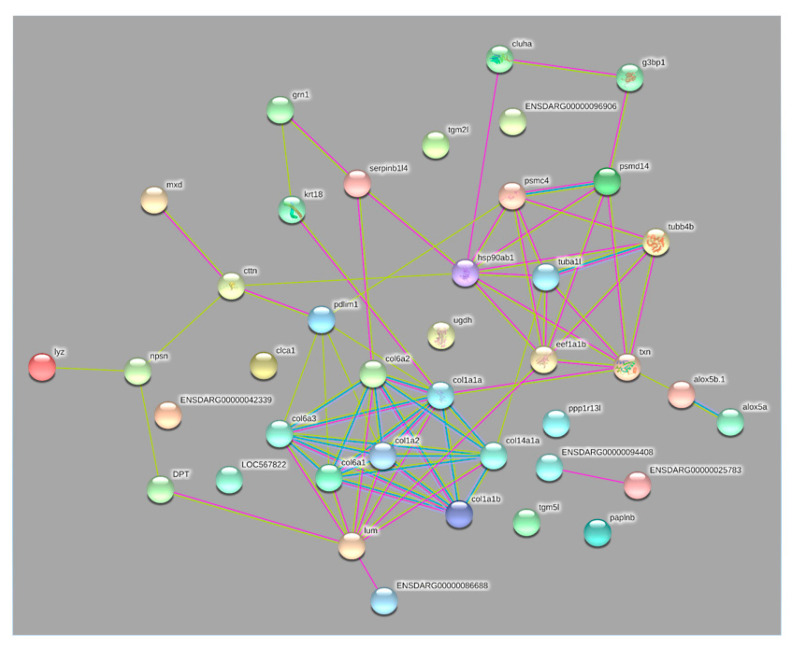
Parasite proteins showing protein-protein interaction with carp mucus proteins. The protein-protein interaction network of carp mucus and *Ichthyophthirius multifiliis* identified proteins. Parasite proteins (tuba1 (alpha tubulin), tubb4b (beta tubulin), eef1a1b (elongation factor 1-alpha; EF-1α), psmd14 (26S proteasome regulatory subunit), psmc4 (26S protease regulatory subunit 6B), and hsp90ab1 (heat shock protein 90; hsp90)) are connected to each other and showed protein-protein interaction with carp mucus proteins thioredoxin, ras GTPase-activating protein-binding protein, PDZ and LIM domain protein 1-like, lumican, and collagen alpha-1(XIV) chain-like. In this network, nodes are proteins; the predicted functional associations are shown as lines, and the strength of predicted functional interactions between proteins is shown as the number of lines. The yellow lines show text-mining evidence; the purple lines denote experimental evidence, and the database evidence is shown as light blue lines.

The identified proteins were: tubulins (alpha and beta), elongation factor alpha, 26S proteasome regulatory subunit, 26S protease regulatory subunit 6B, and heat shock protein 90 (Table 1) and were connected to each other and to the previously identified common carp proteins to identify the protein-protein interaction network (Figure 2).

Two KEGG pathways, phagosome and proteasome, were identified among these parasite proteins. Indeed, keratin I cytoskeletal 18 proteins were differentially modulated in common carp after exposure to *I. multifiliis*, pointing to the actions they may perform in the carp immunity when infected [12]. Keratin is a cytoskeletal protein with a key role in cell protection against mechanical and non-mechanical damage. In fish mucus, the pore formation ability of keratin provides antibacterial effects. Keratin turnover is reliant on the ubiquitin-proteasome pathway. In addition, it could be modulated in response to tissue damages due to parasite proteolytic and phagocytic activities during host invasion and development. Further, several lectins and multiple serpins with protease inhibitory effects were previously identified in *I. multifiliis*-infected carp mucus hinting at their involvement in the activation of the lectin pathway, a cascade of serine proteases, and fine tuning of proteolysis [5].

Parasite proteins showed a protein-protein interaction with carp mucus proteins such as thioredoxin, ras GTPase-activating protein-binding protein, PDZ and LIM domain protein 1-like, lumican, and collagen alpha-1(XIV) chain-like (Figure 2). These parasite proteins are apparently involved in motility, virulence, and the general stress response during parasite growth and development after parasite attachment and host invasion.

### 2.1. The 26S Proteosome

The 26S proteosome complex and 26S protease regulatory subunit 6B were identified in the infected carp skin mucus. Proteases in parasitic protozoa have drawn much attention as prospective drug targets owing to their crucial functions in growth and pathogenicity, and because of the possibility of constructing specific inhibitors [15,16,17,18]. Two cathepsin L cysteine proteases (Icp1 and Icp2) of the C1 papain peptidase family have been described in *I. multifiliis* and suggested to be involved in the disease progression. The cathepsin L cysteine proteases were found to be differentially modulated between all life-stages of *I. multifiliis* and likely involved in host-pathogen interactions [19]. Parasitic protozoa proteases have been shown to play important roles in the overwhelming of host cells and development, encystment and excystment, cytoadherence, and stimulation and evasion of host immune responses, as well as catabolism of host proteins for a nutrient purpose [20,21]. In fish pathogenic ciliates, proteases are key to overcome host immune defenses and lysis of host cells [19] and the marine ciliate *Philasterides dicentrarchi* [22]. Fish parasites generate proteolytic enzymes to lyse collagen and other structural particles to induce deterioration of external epithelia to weaken host defenses [12,22]. In fact, the prevalent increased upregulation of cathepsin L cysteine protease was seen in the infectious stages [19,23]. The proteolytic protein collection (degradome) of the *I. multifiliis* consists of 254 protease homologs, approximately 3.1% of the proteome. The massive retention of duplicates 39 ubiquitin carboxyl-terminal hydrolase family members and 15 members of the threonine proteases occurred due to extensive gene duplication events. This mirrors the fundamental function of the proteasome system in *I. multifiliis* regulating cell-cycle and stress responses [24].

In ciliates, during cell differentiation and transition, prompt alterations in the morphology of cell structure are accompanied with the lysis of numerous proteins. The evidence is increasing that the proteasome system plays crucial role in the degradation of proteins. Further, the 26S proteasome non-ATPase regulatory subunit gene expression was significantly upregulated during cell differentiation of the freshwater ciliate *Pseudourostyla cristata* [25]. Indeed, the *I. multifiliis* 26S proteosome complex and the 26S protease regulatory subunit 6B proteins were identified in infected carp mucus samples. This highlights the importance of these molecules for the regulation of the degradation process of host cell proteins essential for host invasion.

The protein-protein interaction network presented the *I. multifiliis* 26S proteasome connected to carp mucus thioredoxin and ras GTPase-activating protein-binding protein.

As a component of the antioxidant system in living cells, thioredoxins are key for the regulating of redox potential. The increased value of thioredoxin-like isoform X2 protein of *I. multifiliis* observed in carp was aimed at reducing oxidative stress to protect carp against the proteolytic activity of *I. multifiliis* [5]. Indeed, in the current study, the protein-protein interaction network presented the *I. multifiliis* 26S proteasome connected to ras GTPase-activating protein-binding protein agreeing with the previous finding. Ras GTPase-activating protein-binding protein was also augmented in infected common carp mucus samples. This protein was suggested to activate an immune response in carp against *I. multifiliis* [5]. Ras-related proteins are involved in signal transduction, the regulation of TCR signaling, T cell cytoskeletal reorganization, T cell migration, and T cell apoptosis [26]. The protein-protein interaction network displayed 26S proteasome connected to carp mucus thioredoxin and ras GTPase-activating protein-binding protein and likely activates signal transduction and triggers immune defense in carp.

Further, in the current study, the protein-protein interaction network shows that the 26S protease regulatory subunit 6B of *I. multifiliis* connects to PDZ and LIM domain protein 1-like. LIM proteins have been observed to alter NF-κB-mediated signaling in the cytoplasm. It has been reported that PDLIM1 sequestered p65 subunit of NF-κB in the cytoplasm and inhibited its nuclear translocation in an IκBα-independent way [27]. In an earlier investigation, Saleh et al. (2018) observed that the abundance of PDZ and LIM domain protein 1-like was highly increased in common carp, suggesting a suppressive role in the carp immune response once exposed to *I. multifiliis*. This protein seems to trigger the activation of the PDZ and LIM domain protein 1-like aiming at suppressing common carp immune defense as a part of the *I. multifiliis* invasion strategy.

### 2.2. Elongation Factor Alpha

The parasite elongation factor alpha (EF-1α) was identified in the skin mucus of *I. multifiliis*-exposed carp mucus samples. Given that EF-1α has a distinct role in protein synthesis, it is involved in the whole course of the heat shock response in mammalian cells. EF-1α is among the best conserved and highly abundant proteins in the eukaryotic cell and was reported to contribute to the activation of heat shock transcription factors (HSFs) [28]. HSFs sustain protein homeostasis via modulating the expression of heat shock proteins, particularly in stress conditions [29]. Upon stress, EF-1α rapidly activates the transcription of heat shock proteins. This process is affected when EF-1α is inhibited [30]. The identified EF-1α *I. multifiliis* in infected carp mucus purposes its involvement in the activation of HSFs during host invasion.

When analyzing the proteome of the protozoan parasite *Leishmania donovani* exosomes, many proteins including EF-1α and heat shock protein 90 (hsp90) previously reported to enter the cytosol of leishmania-infected macrophages were cargos of these exosomes [31]. Leishmania EF-1α is a part of the exosome and can be transported to macrophage cytosol. It interacts with host Src homology region 2 domain-containing phosphatase-1 (SHP-1), resulting in a deactivated macrophage phenotype. Thus, EF-1α has been considered as a novel virulence factor in *L. donovani*. In ciliates, EF-1α has been shown to interact with cytoskeletal proteins, including tubulin and actin, in numerous organisms [32]. Ciliates have a distinctive cytoskeletal organization that primarily consists of microtubules. In *Tetrahymena*, EF-1α has been reported to bind to F-actin [33]. It has been demonstrated that EF-1α directly binds to β-tubulin [34]. EF-1α appears to have numerous functions such as the translation of mRNA and tubulin binding.

The protein-protein interaction network shows that elongation factor alpha connects to carp mucus lumican and thioredoxin proteins. Lumican controls collagen fibrillogenesis, which supports keeping and maintaining clear corneas as well as stimulating corneal epithelial tissue repair and promoting the structure of numerous other connective tissues such as sclera and skin and as a chemokine gradient maker. It has been reported that lumican contributes to bacterial lipopolysaccharide distinguished by the Toll-like receptor 4 signaling pathway and innate immune response [35]. The abundance of lumican protein was significantly decreased, correlating with the infection and growth of *I. multifiliis*, suggesting that it has a role in immune response and wound healing [12]. Thioredoxins are pivotal in the antioxidant system and a key regulator of redox potential in living cells. The increased value of thioredoxin of *I. multifiliis* observed in carp samples was aimed at reducing oxidative stress to defend carp against the harmful effects of *I. multifiliis* [5]. *I. multifiliis* elongation factor alpha was connected to carp mucus lumican (downregulated) and thioredoxin (increased) proteins likely to affect the carp immune response as a part of a host-manipulating strategy.

### 2.3. Tubulins

*I. multifiliis* contains 9 of the 14 highly conserved core proteins associated with centriole and basal body biogenesis and function [24].

Alpha and beta tubulins of *I. multifiliis* were identified in the infected carp skin mucus.

Motility has been reported to be particularly essential for mucus colonizers such as *V. salmonicida*, as this is favorable in viscous environments such as mucus [36]. A high number of proteins was differentially regulated and uniquely abundant in the theronts but not in the trophont stages of *I. multifiliis* [9]. Among these, proteins correlating with and associated with motility were highly abundant in the theronts’ stages, which reflect the critical need of these parasite stages for rapid and continued motility concomitant with host finding and the infection process [37,38,39].

In the current study, alpha and beta tubulins of *I. multifiliis* were identified in the infected carp skin mucus. This reflects the critical need of *I. multifiliis* for proteins associated with movement during host invasion to ensure rapid and continued motility associated with host finding and the infection process. Additionally, the presented protein-protein interaction network demonstrates that tubulin alpha connects to carp mucus collagen alpha-1(XIV) chain-like and thioredoxin proteins, and tubulin beta connects to thioredoxin.

In fact, the abundance of 11 collagen-alpha family members including collagen alpha-1(XIV) chain-like was extremely reduced in a process of the massive degradation of collagen [12]. This was likely aimed at tissue remodeling, wound healing to concur with injures, and tissue damage of common carp during *I. multifiliis* attachment, movement, and growth. In addition, the increased value of *I. multifiliis* thioredoxin in common carp mucus was likely aimed at reducing oxidative stress during parasite movement and development.

### 2.4. Heat Shock Protein 90

In the present study, hsp90 of *I. multifiliis* was detected in carp skin mucus samples. Heat shock proteins are acknowledged virulence factors in a number of bacteria such as *Vibrio salmonicida* and *Salmonella typhimurium*. A 66 kDa hsp of *S. typhimurium* was reported to be needed for adhesion to mucosal cells [40]. In *V. salmonicida*, the hsps DnaK and GroEL were significantly induced in response to skin mucus [36]. Hsps were also suggested to support the survival of bacteria in their hosts [41]. Heat shock response is a strongly regulated and coordinated response that is indispensable for cell survival under stress. While many hsps mRNAs are present in only very low amounts in unchallenged conditions, their synthesis, stability, and translation increase considerably upon stress [42]. The hsp90 of *Leishmania donovani* plays a key role in homeostasis control and the development of this protozoan parasite. It is also involved in cell cycle control and the cellular stress response [29]. The molecular interaction mediated by hsp90 was suggested to be involved in regulating cortical patterning in *Tetrahymena*, and hsp70 and hsp90 were distinguished in the cilia of *Tetrahymena* [43,44]. Hsp90 chaperones are implicated in intracellular morphogenesis, and hsp90 was found in centrosomes in *Drosophila* and mammalian cell lines and in the basal body region of developing *Drosophila* sperm [45]. In *Tetrahymena thermophila*, at 41 °C, the binding of hsp82p with tubulin at a high temperature (stress) was observed after isolation of an immunoprecipitated hsp82p-hsp73p-tubulin complex from a sucrose density gradient [43]. At the cellular level, both monoclonal and polyclonal antibodies against hsp82p produced general cytoplasmic staining. However, monoclonal antibodies against a 12-amino acid synthetic peptide prepared from a fragment of hsp82p amino acid sequence similarly stained ciliary basal bodies more intensely than the cytoplasmic background [43]. The binding of hsp82p with cortical microtubules occurs at sublethal elevated temperatures (stress) when this protein becomes more abundant and builds complexes with soluble tubulin and with the increased hsp70. This is likely a mechanism for the protection of many proteins in heat-stressed cells [46].

Many genes were differentially expressed among all three life-cycle stages of *I. multifiliis* [23]. Numerous identified genes have a role in cell structure, cell regulation and genes of protein assembly, folding, and translocation, such as hsp70 and hsp90. Additionally, several transcripts comprised cell structural and regulatory proteins including tubulins. Further, hsps are dominant antigens in the immune response to a variety of pathogens. In a previous study, as an immobilization antigen vaccine adjuvant, heat shock protein 70 showed a high protection in fish against *Cryptocaryon irritans* [47]. It has been also reported that tomonts from vulnerable carp were immobilized in in vitro assays when treated with the serum or mucus of immune fish [48]. Immobilization of *I. multifiliis* by humoral elements in the blood and mucus points to a prospective mechanism of host immunity. Antibodies in the mucus likely hinder entering of the parasite into the host, and re-infection is impeded because of possible direct antibody binding and complement activation, or due to antibody-dependent cell-mediated activities [48]. The identification of serum and cutaneous mucosal antibodies recognizing i-antigen has been reported to be associated with effective immune responses when exposed to *I. multifiliis* [49]. Hence, it was anticipated that vaccination against *I. multifilliis* could be achieved. However, to date, there is no cost-effective vaccine available against *I. multifiliis*.

In the current study, *I. multifiliis* hsp90 protein was detected in infected common carp mucus. Hsp90 in *I. multifiiis*-infected carp mucus samples suggests that it builds complexes with tubulin and hsp70 (highly induced in theronts) in a mechanism for protection of a large number of proteins due to stress during host invasion and growth, agreeing with previous reports in *Tetrahymena* [48]. The protein-protein interaction presented hsp90 connected to carp mucus src substrate cortactin-like, leukocyte elastase inhibitor, clustered mitochondria protein homolog, and thioredoxin proteins. Indeed, the carp Src substrate cortactin-like protein was modulated in response to *I. multifiiis* [12]. Cortactin controls actin assembly by activating actin polymerization. Cortactin was identified as the substrates of Src family kinases. Damage to the surface of intact articular cartilage stimulates Src-like kinases, along with MAPKs and IKK, which regulate o NF-κB [50]. Sea lice infection leads to the presence of cleaved fragments from actin and transferrin, suggesting the proteolytic activity of the parasite [11]. The introduction of *L. major* to fibroblasts l caused inhibition of cortactin [51]. The modulation of cortactin in infected carp mucus likely stimulates NF-κB, directed at decreasing inflammatory responses and tissue destruction triggered by *I. multifiliis*.

LEIs belong to the serpins family of proteins, likely stimulated by invading pathogens and have a role in the inhibition and alteration of protease activity directed at reducing host tissue injuries, inflammatory reactions, and apoptosis caused by damaging pathogens [5,52]. Carp LEI proteins increased in response to *I. multifiliis* exposure were proposed to play a role in the inhibition of endogenous proteases to protect leucocytes from degradation and to limit the effects of exogenous proteases of *I. multifiliis* produced during infection [5]. We suggest that carp LEIs are stimulated and activated by *I. multifiliis* hsp90, and hence it might be useful as a vaccine candidate/adjuvant as these molecules prevent leucocytes from degradation and are essential for their survival. The abundance of clustered mitochondria protein homolog (CLUH) and thioredoxin proteins was induced aiming to coordinate the carp immune response and cope with oxidative stress and adverse effects caused by this ciliate.

## 3. Materials and Methods

### 3.1. Ethics Statement

The experiments have been approved by the Animal Experimentation Ethics Committee of Vienna University of Veterinary medicine (BMWFW-68.205/0051-WF/V/3b/2016). The experiments were performed in accordance with relevant guidelines and regulations.

### 3.2. Common Carp and Collection of Skin Mucus

The details of the common carp experimental setup have been described in our earlier study [12]. Prior to infection, common carp (11 ± 1 cm) were distributed between 6 aquaria, 6 fish per aquarium. There were two groups, exposed and non-exposed control. The fish were exposed to *I. multifiliis* by cohabitation with naturally infected giant gouramis (*Osphronemus goramy*) obtained from a pet store—a method that mimics natural exposure. The giant gouramis were certified as free from *Aphanomyces invadans* and the Epizootic Haematopoietic Necrosis Virus. Examinations of the giant gouramis did not reveal the incidence of any other ectoparasite or signs of a secondary bacterial infection. At 1 and 9 days post-exposure (dpe), common carp (*N* = 3) from each of the infected and non-infected control groups were anaesthetized using ethyl 3-aminobenzoate methanesulfonate (Sigma, Darmstadt, Germany) (MS-222; 100 mg/L). Sterile, glass slides were used to collect mucus from fish skin, while avoiding blood contamination, and excluding the ventral body surface close to the anal pore, to prevent fecal contamination. The collected mucus was transferred into 1.5 mL microcentrifuge tubes, immediately placed on ice, then snap-frozen in liquid nitrogen and stored at −80 °C for proteomic analysis.

### 3.3. Protein Extraction, Separation, and In-Gel Digestion

Mucus samples were solubilized in a denaturing lysis buffer (7 M urea, 2 M thiourea, 4% CHAPS, and 1% DTT) containing a mammalian protease inhibitor cocktail. The samples were subjected to sonication on ice, and supernatants were collected by centrifugation. The protein concentration of each sample was measured using the Pierce 660 nm Protein Assay according to the manufacturer’s instructions. The samples were subjected to electrophoresis on 10% SDS-PAGE in biological and technical triplicate, and the gels were stained with silver staining. Protein bands were excised from the gels and were reduced and alkylated [13]. In-gel digestion was performed using trypsin (20 ng/μL) at 37 °C for 8 h according to [14]. Afterwards, peptides were extracted, dried, and redissolved in 0.1% trifluoroacidic acid.

### 3.4. Mass Spectrometry

Tryptic peptides were separated on a nano-HPLC Ultimate 3000 RSLC system (Dionex, Sunnyvale, CA, USA) and analyzed with a high-resolution hybrid triple quadrupole time of flight mass spectrometer (TripleTOF 5600+, Sciex, Framingham, MA, USA). Details of the LC–MS/MS procedure were described earlier [12]. Acquired raw data were processed with ProteinPilot Software version 5.0 (Sciex). The database consisted of NCBI and UniProt entries of the following taxonomies: *Ichthyophthirius multifiliis* (taxonomy id: 5932) and the common Repository of Adventitious proteins (available online: ftp://ftp.thegpm.org/fasta/cRAP/crap.fasta (accessed on 5 April 2021)). Mass tolerances in MS mode were 0.05 Da, and 0.1 Da in MSMS mode, for the rapid re-calibration search, and 0.0011 Da in MS and 0.01 Da in MSMS mode for the final search. The following parameters were used: trypsin digestion, cysteine alkylation with iodoacetamide, and rapid ID. The false discovery rate analysis was set to <1% on the protein and on the peptide level.

Proteomics data have been deposited in the ProteomeXchange Consortium via the PRIDE (Cambridge, UK) partner repository with the dataset identifier PXD011148.

To determine the protein-protein interaction network of carp mucus and *I. multifiliis* proteins, amino acid sequences of previously identified mucus proteins [5,12] and 6 *I. multifiliis* proteins were analyzed using homologs of *Danio rerio* by using STRING software (version 11.0). A representation of the protein-protein interaction network was performed at confidence score 0.15 in the text-mining, experiment, and databases.

## 4. Conclusions

The identification of these six *I. multifiliis* proteins in infected common carp skin mucus suggests their possible role in the host invasion strategy of this ciliate to conquer host immune defenses effectively. The identification of hsp90 during infection hints that it is a suitable drug and vaccine target and may function as an antigen vaccine adjuvant in a way similar to that of *C. irritans*. Further, in addition to hsp90, which is likely activated by eEF through HSFs, the identification of b-tubulins (important for ciliary function) suggests roles in mobility and viability of the parasite as well as in homeostasis control and the cellular stress response during host invasion and growth, as previously suggested for other protozoan parasites. The identified proteins are implicated in the heat shock response, suggesting they are likely key for parasite development, virulence, and pathogenicity. However, functional experiments and host-parasite interaction studies are required to confirm and elucidate the precise mechanisms of *I. multifiliis* pathogenesis. This may help us discover potential vaccine and drug targets, which could support the management of ichthyophthiriosis in aquaculture.

## Figures and Tables

**Table 1:** Identified *Ichthyophthirius multifiliis* proteins in the skin mucus of infected common carp.

UniProt Accession Number	Protein	Confident Peptides	Coverage (%)	Function
G0QTP1_ICHMG	Tubulin alpha chain	12	25.4	Microtubule-based process
G0QMB0_ICHMG	Tubulin beta chain	9	33.0	Microtubule-based process
G0QQR6_ICHMG	Elongation factor 1-alpha	8	18.2	Translation elongation factor activity
G0R170_ICHMG	26S proteasome regulatory subunit	3	5.3	Protein catabolic process
G0QY27_ICHMG	26S protease regulatory subunit 6B/AAA domain-containing protein	3	10.1	Protein catabolic process
G0QRA5_ICHMG	Heat shock protein 90/HATPase_c domain-containing protein	3	5.8	Protein folding

## Data Availability

The data presented in this study are available on request from the corresponding author.
